# The Role of the *S40* Gene Family in Leaf Senescence

**DOI:** 10.3390/ijms18102152

**Published:** 2017-10-16

**Authors:** Muhammad Jehanzeb, Xiangzi Zheng, Ying Miao

**Affiliations:** The Center for Molecular Cell and Systems Biology, Fujian Provincial Key Laboratory of Haixia Applied Plant Systems Biology, College of Life Sciences, Fujian Agriculture & Forestry University, Fuzhou 350002, China; 2141939001@fafu.edu.cn (M.J.); 000q8161021@fafu.edu.cn (X.Z.)

**Keywords:** S40 protein family, senescence, environmental cues

## Abstract

Senescence affect different traits of plants, such as the ripening of fruit, number, quality and timing of seed maturation. While senescence is induced by age, growth hormones and different environmental stresses, a highly organized genetic mechanism related to substantial changes in gene expression regulates the process. Only a few genes associated to senescence have been identified in crop plants despite the vital significance of senescence for crop yield. The *S40* gene family has been shown to play a role in leaf senescence. The barley *HvS40* gene is one of the senescence marker genes which shows expression during age-dependent as well as dark-induced senescence. Like barley *HvS40*, the Arabidopsis *AtS40-3* gene is also induced during natural senescence as well as in response to treatment with abscisic acid, salicylic acid, darkness and pathogen attack. It is speculated that rice *OsS40* has a similar function in the leaf senescence of rice.

## 1. Introduction

The last phase of leaf development prior to cell death is leaf senescence. The degradation of cellular materials and their remobilization to develop leaves and emerging seeds characterizes the process [1,2]. In cereal crops, a close relationship exists between leaf senescence and the seed maturation induction process [3,4]. While early senescence caused substantial production loss [5,6], increased yield was shown by stay-green phenotypes in different crops [7]. In the USA and Canada, the yield of *Zea mays* L. (maize) increased significantly due to delayed leaf senescence [8,9] while the yield of soybean decreased due to precocious leaf senescence [10]. Similarly, the weight of seeds in the maize plants increased by 15–30% and the yield of grains in rice increased by 24% [11] and 10.3% [12] due to delayed senescence. Leaf senescence directly influences the quality and yield of a crop, and therefore can be manipulated for improving crop quality and increasing the yield [11].

Leaf senescence is an age-dependent process, yet it is induced by various external and internal factors. External factors include biotic factors such as pathogen attack, and abiotic stresses such as salt stress, drought stress, wounding, heat, nutrient stresses, high light and darkness [13,14,15,16,17,18,19]. Internal factors include plant growth hormones and aging. Plant hormones include abscisic acid (ABA), salicylic acid (SA), jasmonic acid (JA), auxin, ethylene, and brassinosteroids (BR) that positively regulate senescence, while cytokinins and polyamines negatively regulate senescence [20,21]. In this sense, the suppression of plant hormones that promote senescence such as ethylene or overproduction of senescence-inhibiting hormones such as cytokinins could delay plant senescence [20,21].

Senescence is a highly organized process that requires the expression of specific genes [22,23], referred to as SAGs (senescence-associated genes), that account for 10% of a plant genome [24,25] during aging. Thousands of genes show differential expression patterns during the onset and development of senescence [25,26]. In Arabidopsis, around 20% of all the genes showed differential expression during leaf senescence [27] and 827 SAGs were found to be upregulated at least three times upon senescence [16]. Transcriptomic studies have revealed a wide range of change in gene-expression during senescence in many plants such as *Arabidopsis thaliana* [26,28], *Triticum aestivum* [29], *Hordeum vulgare* [30], *Medicago truncatula* [31], *Oryza sativa* [32] and *Aspen* [33]. Different transcription factors (TFs) among which transcription factor NAM (no apical meristem), ATAF, CUC (cup-shaped cotyledon) (NAC) and transcription factor in which N-terminal ends contain a conserved WRKYGQR amino acids sequences (WRKY) are the two major groups [25] that activate these SAGs, hence the regulation of a gene expression that encodes a specific, important transcription factor can influence the expression of numerous genes that are involved in plant senescence [34].

**Figure 1:**
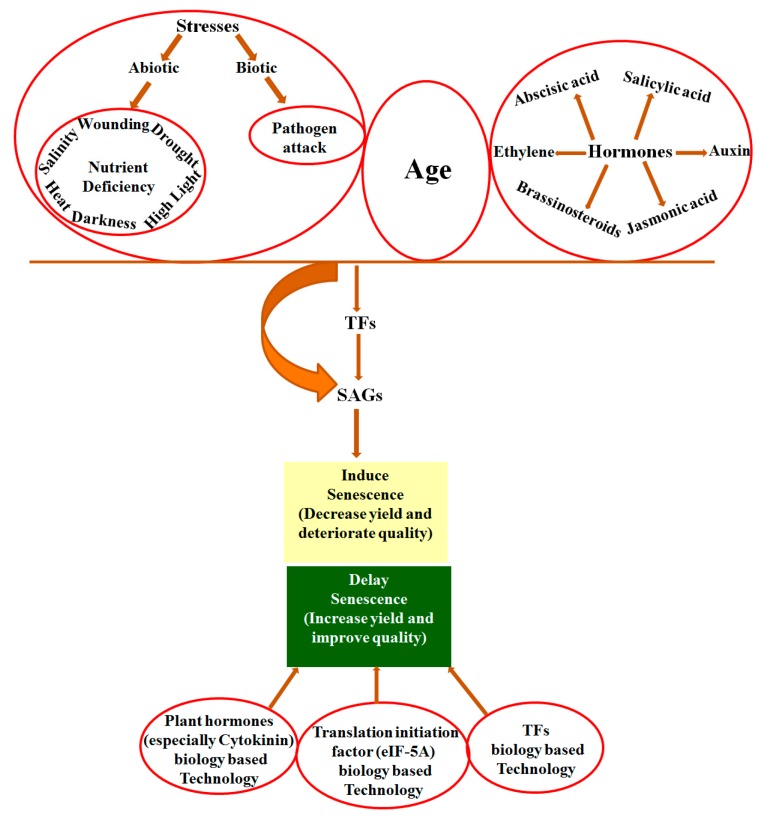
Transcriptional regulation and manipulation of leaf senescence.

Arabidopsis NAP (*AtNAP*), one of the members of the NAC family, is a key transcription factor that plays a regulatory role in leaf senescence. The counterpart genes in other crops such as morning glory [35], *Festuca arundinacea* [36], *Phaseolus vulgaris*, *Oryza sativa* [37] and *Bambusa vulgaris* [38] also showed expression only in non-senescent leaves, but not in young leaves. Moreover, the expression pattern of these homologues was similar to *AtNAP* [36,37,38], which indicates their regulatory role in leaf senescence. The corresponding genes of *AtNAP* showed delayed leaf senescence in rice [39] and maize [40]. Similarly, WRKY transcription factors such as WRKY70, WRKY54 [41], WRKY53 [42], WRKY22 [43], WRKY23 [44], WRKY42 [45], WRKY80 [46] and WRKY6 [47] also play a role in regulating leaf senescence. Genomic pull-down assays identified sixty-three genes as direct targets of WRKY53, including at least six other members of the *WRKY* gene family. This indicates that WRKY53 transcription factors regulate SAGs and act as an upstream control element in a WRKY signaling cascade [42] (Figure 1).

Generally, in cereal crops, an inverse proportion exists between leaf senescence and seed production yields; that is, early senescence causes substantial production loss, and vice versa. It is important to manipulate plant senescence by biotechnology methods to increase crop yields. The *S40* gene family is one of few genes identified as being associated with plant leaf senescence. This review deals with the role of the *S40* gene family in, mainly, barley and rice as well as Arabidopsis. It describes (1) phylogenetic trees; (2) the alteration of gene expression; (3) the subcellular localization of S40 proteins; (4) the nature of *S40* mutants; (5) regulation at the transcriptional level; (6) *cis* elements comparison; and (7) WHIRLY1 protein bindings to the *HvS40* promoter. This compilation of information is thought to be timely and useful for people working with similar kinds of subjects.

## 2. Phylogenetic Tree Construction

S40 belongs to the DUF584 family, containing the DUF584 domain. All members of the DUF584 group shared the sequence GRXLKGR(D/E)(L/M)XXXR(D/N/T)X(I/V)XXXXG(F/I) [48]. This sequence belongs to the C-terminal domain. In barley, only two proteins belong to DUF584 family, of which one is encoded by *HvS40* gene, while in Arabidopsis, fifteen proteins belong to this family [49]. An alignment by Clustal W [50] showed that the proteins can be further divided into five groups on the basis of similarities among the amino acid sequences. Four DUF584 proteins of Arabidopsis and the HvS40 protein belong to the same group, group I. In the remaining eleven of the Arabidopsis DUF584 protein family, three proteins lie in group II, four proteins lie in group III, three proteins lie in group IV, while only one Arabidopsis protein belongs to Group V, which also has the second barley DUF584 protein [49].

**Figure 2:**
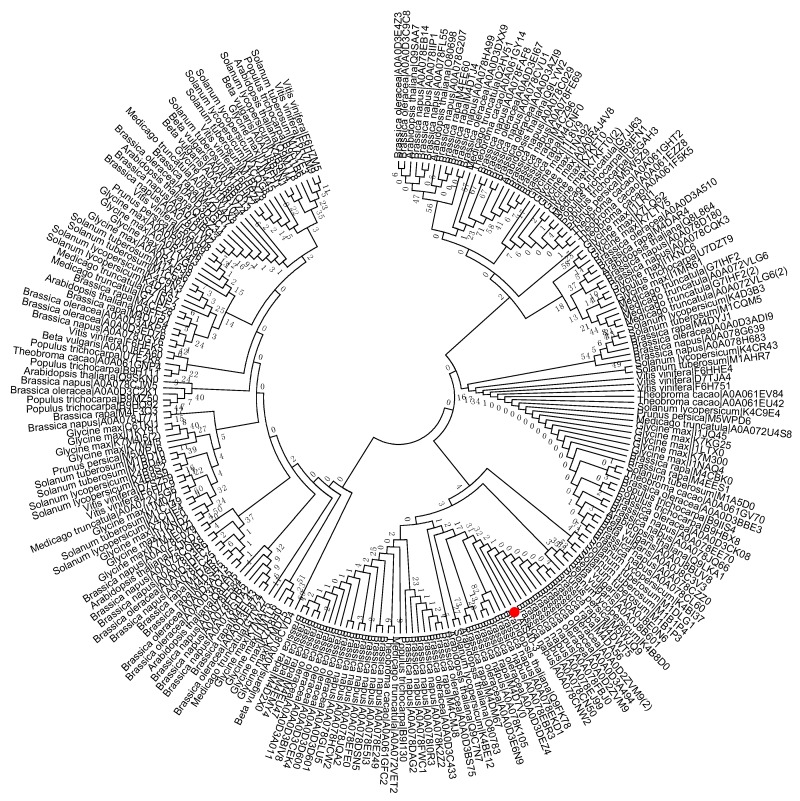
Phylogenetic tree of the *S40* gene family in sequenced genome dicot plants including Arabidopsis thaliana, Beta vulgaris, Brassica napus, Brassica oleracea, Brassica rapa, Glycine max, Medicago truncatula, Populus trichocarpa, Prunus persica, Solanum lycopersicum, Solanum tuberosum, Theobroma cacao, and Vitis vinifera. Red dot indicates the *S40* senescence reference gene, *AtS40-3*. The MEGA 6.0 program was employed to construct a neighbor-joining phylogenetic tree using the JTT model with 1000 bootstrap replicates. The full-length amino acid sequences were aligned using Clustal Omega.

**Figure 3:**
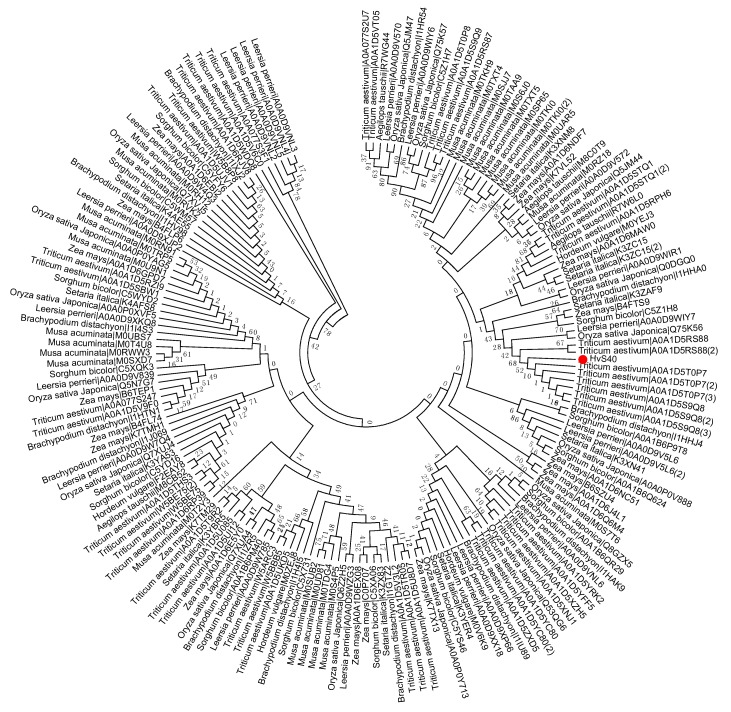
Phylogenetic tree of the *S40* gene family in sequenced genome monocot plants including Aegilops tauschii, Brachypodium distachyon, Hordeum vulgare, Leersia perrieri, Musa acuminate, Oryza sativa Japonica, Setaria italica, Sorghum bicolor, Triticum aestivum, Zea mays. Red dot indicates the *S40* senescence reference gene, *HvS40*. The MEGA 6.0 program was employed to construct a neighbor-joining phylogenetic tree using the JTT model with 1000 bootstrap replicates. The full-length amino acid sequences were aligned using Clustal Omega.

**Figure 4:**
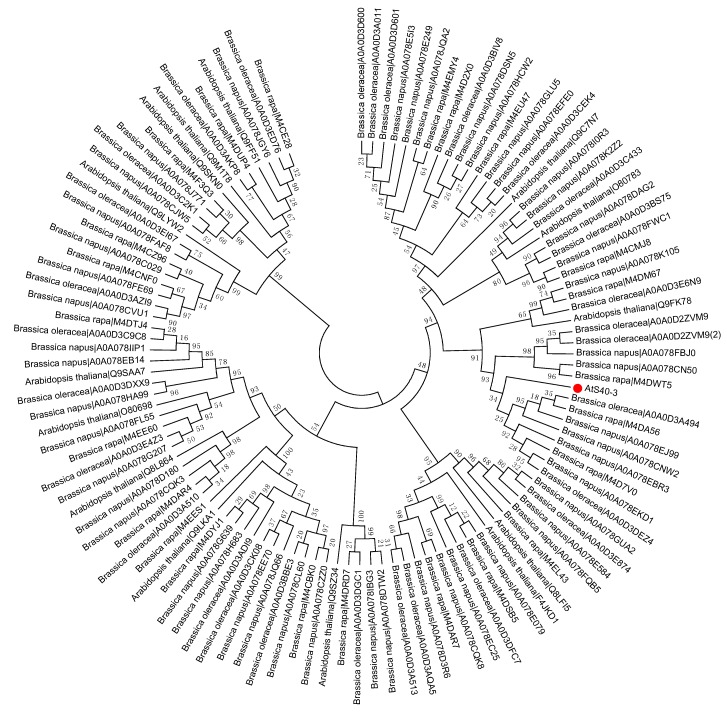
Phylogenetic tree of the *S40* gene family in the sequenced genome *Brassicaceae* family including *Arabidopsis thaliana*, *Brassica napus*, *Brassica oleracea*, and *Brassica rapa*. Red dot indicates the *S40* senescence reference gene, *AtS40-3*. The MEGA 6.0 program was employed to construct a neighbor-joining phylogenetic tree using the JTT model with 1000 bootstrap replicates. The full-length amino acid sequences were aligned using Clustal Omega.

**Figure 5:**
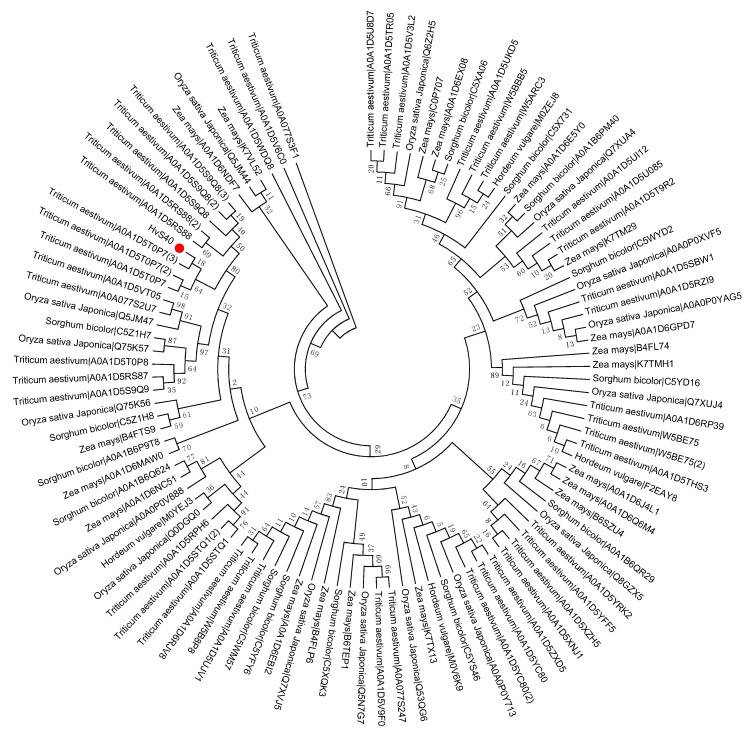
Phylogenetic tree of the *S40* gene family in sequenced genome cereal crop plants including *Oryza sativa Japonica*, *Triticum aestivum*, *Zea mays*, *Hordeum vulgare*, and *Sorghum bicolor*. Red dot indicates the *S40* senescence reference gene, *HvS40*. The MEGA 6.0 program was employed to construct a neighbor-joining phylogenetic tree using the JTT model with 1000 bootstrap replicates. The full-length amino acid sequences were aligned using Clustal Omega.

To study the evolutionary history of S40 proteins, a HvS40 protein sequence was used as a Basic Local Alignment Search Tool (BLAST) query against the available sequenced genome of plants using http://plants.ensembl.org/Multi/Tools/database. The aligned sequences were obtained from dicot plants including *Arabidopsis thaliana*, *Beta vulgaris*, *Brassica napus*, *Brassica oleracea*, *Brassica rapa*, *Glycine max*, *Medicago truncatula*, *Populus trichocarpa*, *Prunus persica*, *Solanum lycopersicum*, *Solanum tuberosum*, *Theobroma cacao* and *Vitis vinifera*, while monocot plants included *Aegilops tauschii*, *Brachypodium distachyon*, *Hordeum vulgare*, *Leersia perrieri*, *Musa acuminate*, *Oryza sativa* Japonica, *Setaria italica*, *Sorghum bicolor*, *Triticum aestivum* and *Zea mays*. When the *HvS40* protein sequence was blasted against the genomes of *Chlamydomonas reinhardtii*, *Chondrus crispus*, *Cyanidioschyzon merolae* strain 10D, *Ectocarpus siliculosus*, *Galdieria sulphuraria*, *Ostreococcus lucimarinus* CCE9901, *Picea sitchensis* and *Thalassiosira pseudonana*, no similar hit was found. The MEGA 6.0 program was employed to construct a neighbor-joining phylogenetic tree using a JTT model with 1000 bootstrap replicates. The full length amino acid sequences were aligned by using Clustal Omega. To identify the HvS40 protein aligned members, four separate trees were constructed in which the first tree includes dicot plants (Figure 2), the second includes monocot plants (Figure 3), the third includes *Brassicaceae* family plants (Figure 4), and the fourth includes cereal crop plants (Figure 5). Among the dicot plants, several members from *Brassica napus*, *Brassica oleracea* and *Brassica rapa* were identified close to AtS40-3, while in monocot, several members from *Triticum aestivum* were identified close to HvS40 that may have similar functions in these plants. Plenty of HvS40 protein-aligned members were found in cereal crops as well as *Brassicaceae* family plants. Sixteen members of the *S40* gene family were found in the rice (*Oryza sativa* Japonica) genome. It seems that the *S40* gene family does not exist in lower organisms such as *Thallophytic*, and does also not exist in *Gymnosperms* plants.

## 3. Alterations in *S40* Genes Correspond to Age-Dependent as Well as Artificially-Induced Leaf Senescence

In barley flag leaves, the expression level of *HvS40* increased simultaneously with chlorophyll degradation and reached the highest level shortly before the last stage of senescence [14]. Thus, *HvS40* gene expression can be used as a sign for age-dependent senescence [51]. In *Arabidopsis*, seven out of eleven genes, *AtS40-1*, *AtS40-2*, *AtS40-3*, *AtS40-4*, *AtS40-5*, *AtS40-6* and *AtS40-7* showed enhanced transcripts levels in senescent leaves compared to non-senescent leaves [49]. When barley leaf segments were exposed to drought stress and treated with hydrogen peroxide, the *HvS40* gene showed a highly upregulated expression level [52], while treatment with mannitol to provoke osmotic stress induced its expression only slightly [53]. Although *HvS40* expression was enhanced by treatment of barley leaf parts with abscisic acid (ABA), salicylic acid (SA), ethylene and jasmonic acid (JA) [54], the response to ABA was highly substantial [55]. Similarly, the expression level of three genes *AtS40-2*, *AtS40-3* and *AtS40-4* were shown to be slightly increased after the treatment of the Arabidopsis plants with salicylic acid (SA) and ABA [49]. *HvS40* expression can be used as a sign for senescence induced by darkness [14] and the gene showed highly upregulated expression levels in barley [56]. The expression level of *AtS40-1*, *AtS40-2* and *AtS40-5* genes were induced after two days of dark incubation while *AtS40-3* and *At-S40-4* showed increased expression only one day after dark incubation [49].

When the leaves of a barley plant were incubated with the pathogen *Pyrenophora teres*, the *HvS40* gene showed a highly induced expression level in the infected leaf segments at the necrotic lesion sites surrounded by chlorotic tissue [54]. Similarly, after the infection of the Arabidopsis plants with the pathogen *Pseudomonas syringae* for only one day, *AtS40-2*, *AtS40-3* and *AtS40-4* also showed increased expression [49].

By using the Bio-Analytic Resource for the plant biology database (http://bar.utoronto.ca/efprice/cgi-bin/efpWeb.cgi), we predicted the expression pattern of the *OsS40* gene family in rice. The sequences for 16 putative *S40* gene family members in rice (*Oryza sativa*) were obtained from the Rice Genome Annotation project (http://rice.plantbiology.msu.edu/) that were labeled as *OsS40-1* to *OsS40-16*. During age-dependent senescence, the expression patterns of *S40-1*, *S40-13* and *S40-16* were shown to be slightly upregulated, while *S40-4*, *S40-7*, *S40-14* and *S40-15* showed significant upregulation. *S40-1*, *S40-4*, *S40-6*, *S40-7*, *S40-9*, *S40-13*, *S40-15* and *S40-16* showed significant upregulation to salt stress while *S40-5*, *S40-6*, *S40-7*, *S40-9* and *S40-14* showed significant upregulation to drought stress. None of the genes showed upregulation during cold stress (Table 1).

## 4. Subcellular Localization

When the barley *HvS40* 15.4 kD small protein was fused to β-glucuronidase (GUS) [54] or to green fluorescent protein (GFP) [48], it was found to be located in the nucleus [54]. Due to the inclusion of two nuclear localization signals (NLS) in the sequence, this is expected. The theoretical molecular weights of the seven candidate proteins encoded by the *AtS40* genes ranged from 12 to 25 kD. The six candidate proteins showed accumulation only in the cytoplasm while the *AtS40-3*-GUS fusion protein was localized both in the cytoplasm and nucleus. After transformation with the *AtS40-3*-GUS construct, GUS activity distribution in the cell [49] showed similarity to GUS activity distribution after transformation with the *HvS40*-GUS fusion construct [54].

The putative subcellular localization of S40 proteins in rice was predicted using the publicly available web services YLoc, WoLF PSORT, DISTILL 2.0, LOCALIZER 1.0, BaCelLo and Euk-mPLoc-2.0. A dominant number of prediction programs predicted the localization of *OsS40-1*, *OsS40-2*, *OsS40-3*, *OsS40-5*, *OsS40-6*, *OsS40-7*, *OsS40-9*, *OsS40-14* and *OsS40-16* in the nucleus while *OsS40-12* and *OsS40-13* localization were predicted to be in the chloroplast. The prediction programs predict different localizations of *OsS40-4*, *OsS40-8*, *OsS40-11* and *OsS40-15* as shown in the Table 2. Although the prediction tool shows a high ratio of accuracy, the exact localization must be tested via experimentation.

## 5. Natural Leaf Senescence Is Delayed and SAGs Are Repressed in the *S40* Mutant

In *Arabidopsis* wildtype plants, the *AtS40-3* gene showed 16 times higher expression levels in senescent leaves compared to non-senescent ones during natural senescence. While in the *AtS40-3a* mutant, the *AtS40-3* gene expression level was similar in both non-senescent rosettes and senescent ones indicating that the gene was disabled for senescence-specific induction. The results showed a delayed senescence in the *AtS40-3a* mutant in comparison to the wildtype [49]. The expression level of the *WRKY53* gene, which is a marker of early changes in gene expression during leaf senescence, was significantly down-regulated in the mutant in comparison to the wildtype. Similarly, the expression level of the *SAG12* gene, which is a marker of late changes in gene expression, was clearly decreased in the *AtS40-3a* mutant in comparison to the wildtype at all stages of senescence [49]. The expression analyses in both the wildtype and mutants propose that the *AtS40-3* gene acts as an activator of downstream SAGs, *SAG12* and *WRKY53*.

During dark-induced senescence, the kinetics and expression level of SAGs changed in the *AtS40-3a* mutant in comparison to the wildtype. In the wildtype, the expression level of the *SEN1* gene, SAG [57] was enhanced about 375-fold after one day of darkness and thereafter decreased. While in the *AtS40-3a* mutant, *SEN1* expression increased only 100-fold after one day of dark incubation and increased further. In the wildtype, the expression level of the *SAG12* gene was increased after dark incubation, which increased further with time in darkness. While in the *AtS40-3a* mutant, the *SAG12* gene showed almost no expression after the first two days of dark incubation, and after three days of dark incubation it reached almost half of the expression level noticed in wildtype plants [49]. These results indicate that the *AtS40-3* gene positively regulates senescence in natural light as well as dark conditions. The development of *Hvs40* mutants for the phenotypic analysis as well as for the analysis of barley SAG expression in the mutants will improve the understanding of the senescence-associated role of *HvS40*.

## 6. Senescence-Related Expression of the *S40* Gene Shows Regulation at the Transcriptional Level

Senescence processes were shown to be induced during dark incubation which is partially reverted after subsequent re-illumination. *HvS40* was shown to accelerate after one day of dark incubation, which was decreased to unnoticeable levels after the exposure of plants to light for one day [56,58]. During dark treatment, the *HvS40* gene was shown to be transcribed before the onset of senescence by the hybridization of nuclear run-on (NRO) transcripts. However, during dark-induced senescence, the transcription rates significantly increased and then declined when the senescence process was reversed by re-illumination [55]. The transcription rate of 19 barley SAGs, which showed increased levels of expression on exposure to darkness, were examined in parallel for comparison by NRO transcription assays. During the dark-induced senescence, five of 19 genes showed a higher rate of transcription [59]. By comparing the *HvS40* gene promoter sequence with two other barley SAGs, one SAG (*HvSD1*) showed regulation at the transcriptional level only in leaves exposed to darkness while the other SAG (*HvSD8*) did not show regulation at the transcriptional level [55]. The *HvS40* gene also showed increased transcription in the nuclei of senescent flag leaves [59], whereas *HvSD1* transcription was not increased under the same conditions. Taken together these results illustrate that during senescence, the upstream regulatory components shared by various types of senescence regulate *HvS40* gene expression at the transcriptional level. The regulation level of the senescence-related *AtS40-3* gene in Arabidopsis is yet to be examined.

## 7. *Cis* Elements Comparison in the Promoters of *S40* Genes in Barley, Arabidopsis and Rice

To reveal *cis* elements that might be involved in the initiation of *HvS40* gene transcription during natural and dark-induced senescence and to gain insight into elements related to developmental senescence, the promoter of the *HvS40* was compared with the promoters of *S40* genes in barley, Arabidopsis and rice. Along with many myelocytomatosis (MYC, CANNTG) and myeloblastosis (MYB, ACGTG), several dehydration-responsive elements/C-repeats (DRE/CRT, A/GCCGAC) and abscisic acid responsive elements (ABRE, ACGTG) recognition sequences were found in *HvS40* promoter sequence using in silico analyses, while only a few of the latter motifs were found in *HvSD1* and *HvSD8* promoters (Table 3). In the *HvS40* gene upstream region, among the most abundant motifs, several DNA-binding with one finger (Dof) binding sites (AAAG) and many light-regulated elements (LREs) along with embryo-specific expression motifs and pathogen-responsive elements were also identified. Dof binding sites and pathogen-responsive elements were identified repeatedly in the *HvSD1* gene promoter compared to the *HvS40* gene promoter (Table 3). All the three analyzed promoters contained several W-box elements. W-boxes were shown to bind *WRKY* TFs [60,61].

Two prominent motifs, W-box elements ([T]TGAC[C/T]) and inverted repeat motifs (IR2, TGTCA) were found only in the *HvS40* promoter. The first motif (MI) that contained a W-box and an IR2 motif was situated 613–631-bp upstream of the transcription initiation site, while the second motif (MII), containing a W-box dimer motif, was located 401–420-bp upstream from the transcription initiation site [55]. The MI motif was shown to be highly identical to the elicitor response element (ERE) element found in the promoter of the *PR10a* gene in potato. In the potato, during pathogen attack, binding of *StWHY1* TF to an ERE element that consists of a W-box and an IR2 element was observed [62,63]. The MII motif was found to be highly similar to a W-box dimer motif in the promoter of *SIRK/FRK1* gene of *Arabidopsis* (*At2g19190*) that encodes a receptor kinase, which shows upregulated expression upon pathogen challenge, as well as during leaf senescence [64]. Moreover, the binding of *WRKY11* and *WRKY26* TFs to this W-box dimer motif was also found [61]. This suggests a multidimensional function of the W-box motifs that controls transcription during various developmental as well as environmental conditions by binding various TFs.

Using the phylogenetic analysis, the promoters of the *S40* genes in rice and Arabidopsis that are closest to the *HvS40* gene were analyzed by the plant cis-acting regulatory DNA elements (PLACE) database (http://www.dna.affrc.go.jp/PLACE/). Promoter regions of 1000 bp upstream of *AtS40-3*, *At5g45630*, *At1g29640*, *At2g34340* and *OsS40-1*, *OsS40-2*, *OsS40-7*, *OsS40-14* were analyzed to reveal *cis* regulatory elements. The prominent motif, a deoxyribonucleic acid (DNA) *cis*-regulatory element sequence, (T)TGAC)C/T), which is recognized by the family of WRKY transcription factors (W-box) along with many MYC, MYB, ABRE, and Dof binding sites and a few DRE/CRT recognition sequences were found in the promoter regions of Arabidopsis and rice *S40* genes, as shown in Table 3. The W-box is an element known to be the binding site of WRKY transcription factors [65]; ABRE is an ABA-responsive *cis*-acting element found in many ABA-inducible genes [66,67,68]; and the Dof proteins are a family of plant-specific TFs that includes Dof1, Dof2, Dof3, and PBF [69] Similar function of the *cis* elements are speculated in the regulation of *S40* genes in Arabidopsis and rice.

## 8. The WHIRLY1 Protein Binds to *HvS40* Promoters at the MII Motif Region

The MI and MII motifs (W-box dimer motif) of *HvS40* are separated by a 200 bp sequence stretch. Three DNA–protein complexes with similar mobility were identified (complexes I, II, III) with motifs as well as nuclear protein extracts from young and senescent leaves of barley using electrophoretic mobility shift assays. The complex of intermediate mobility among the three complexes that were formed at MII, which is close to the start codon, was derived solely with nuclear proteins obtained from young leaves. While the complex of intermediate mobility among the three complexes formed at MI was derived solely with nuclear proteins from senescent leaves. By using a biotinylated DNA fragment that contains the MII motif to bind the nuclear proteins from nonsenescent leaves, a protein of 24-kDa was extracted, which was recognized as WHIRLY1 on the basis of the amino acid sequences of three peptides [55].

Recombinant WHIRLY1 was shown to bind with MII as well as the MI motif. A complex of mobility formed at MI with nuclear proteins derived from senescent leaves showed similarity to the one that contained WHIRLY1 at MII in young leaves. This suggests that WHIRLY1 might negatively regulate the *HvS40* gene, which represses the expression of the *HvS40* gene prior to the initiation of senescence, because WHIRLY1 has already been shown as a factor binding to the MII of the *HvS40* promoter only in young leaves. This also suggests that before the onset of senescence, WHIRLY1 might bind to the *HvS40* promoter at different positions, as happens during senescence. It is possible that this alteration in position changes the binding efficiency of the promoter for various TFs. These different TFs that bind to the W-box motifs of MII may include *WRKY* TFs. As in the promoter of the *SIRK/FRK1* gene of Arabidopsis, five nominated *WRKY* TFs that represent the three main WRKY protein groups were found to bind to the W-box dimer motif [61], which has high similarity to the *HvS40* MII motif. Furthermore, various adjacent W-box elements have been reported in various gene promoters. In the case of HvWRKY38 TF in barley, two closely adjacent W-box motifs are essential for efficient DNA binding, while PcWRKY1 tends to have synergistic effects on DNA transcription [70,71]. However, most of the WRKY TFs, might bind as a monomer to only one of the two W-box regions contained within the dimer motifs [61]. For example, it is reported that for the effective DNA binding of HvWHIRLY1 on motif II (MIII) at the *HvS40* promoter region, two unaltered W-box motifs are required. These findings clearly show that HvWHIRLY1 TF has a lower binding capability to the W-box motifs as compared to the WRKY TFs [55]. It is reported that there is a higher expression level of *WRKY* TFs during pathogen attack and during senescence [65], this may be due to altered gene expression resulting from the replacement of the WHIRLY1 TF from the promoter of *HvS40*. However, a chromatin immunoprecipitation comprised of HvWHIRLY1-specific antibody is needed to investigate whether the change of the position of WHIRLY1 at the promoter region of *HvS40* during development alters its expression. Furthermore, the development of transgenic plants with different levels of WHIRLY1 TF is also needed to explore the effect of WHIRLY1 TF on *HvS40* gene expression.

## 9. Conclusions

Regarding the potential use of senescence for improving yield, much of the fundamental knowledge of the regulation of senescence has been tested in crop species such as those with stay-green traits [72] and *pSAG12:IPT* technology [73]. Further elucidating the genes related to senescence will expand the understanding of leaf senescence regulation at the molecular level. The *S40* gene family showed enhanced expression levels during natural as well as stress-induced senescence in barley and Arabidopsis. The *AtS40-3* gene positively regulates senescence in natural light as well as in dark conditions. Upstream regulatory components shared by the various types of senescence regulate *HvS40* gene expression at the transcriptional level during senescence. The single-stranded DNA-binding protein WHIRLY1 binds to the *HvS40* promoter at the MII motif region. The *S40* gene family plays a regulatory role in leaf senescence in *Arabidopsis* and barley plants. The predicted upregulated expression pattern of some members of the *S40* gene family in rice insinuate its regulatory role in rice leaf senescence. Different parameters still need to be studied to gain insight into the complex regulation of senescence by the *S40* gene family, particularly in the model plant, *Arabidopsis*. The development of transgenic plants and further efforts for detailed functional analyses of the *S40* gene family in rice, wheat and other crops will explore the precise mechanism of function of the *S40* gene family related to senescence.

## Figures and Tables

**Table 1:** Predicted expression level of *OsS40* genes under natural and stress-induced senescence (http://bar.utoronto.ca/efprice/cgi-bin/efpWeb.cgi).

*S40* Genes	Age Dependent Senescence	Salt Stress	Drought Stress	Cold Stress
*OsS40-1*	Slightly increased	Increased	No change	No change
*OsS40-2*	No change	No change	No change	No change
*OsS40-3*	No change	No change	No change	No change
*OsS40-4*	Increased	Increased	No change	No change
*OsS40-5*	No change	No change	Increased	No change
*OsS40-6*	No change	Increased	Increased	No change
*OsS40-7*	Increased	Increased	Increased	No change
*OsS40-8*	No change	No change	No change	No change
*OsS40-9*	No change	Increased	Increased	No change
*OsS40-10*	No change	No change	No change	No change
*OsS40-11*	No change	No change	No change	No change
*OsS40-12*	No change	No change	No change	No change
*OsS40-13*	Slightly increased	Increased	No change	No change
*OsS40-14*	Increased	No change	Increased	No change
*OsS40-15*	Increased	Increased	No change	No change
*OsS40-16*	Slightly increased	Increased	No change	No change

**Table 2:** The putative subcellular localization of *S40* protein in rice *.

Loci Number in Genome	Gene Name	Yloc (http://abi.inf.uni-tuebingen.de/Services/YLoc/webloc.cgi)	WoLF PSORT (https://wolfpsort.hgc.jp/)	DISTILL 2.0 (http://distillf.ucd.ie/distill/)	LOCALI-ZER 1.0 (http://localizer.csiro.au/)	BaCelLo (http://gpcr.biocomp.unibo.it/bacello/pred.htm)	Euk-mPLoc 2.0 (http://www.csbio.sjtu.edu.cn/bioinf/euk-?multi-2/)
Loc_Hv FI496079	*HvS40*—Nucleus (Krupinska et al. 2002) [54]	Nucleus	Nucleus	Nucleus	Nucleus	Nucleus	Mitochondria. Nucleus
LOC_Os05g45450	*OsS40-1*	Nucleus	Nucleus	Nucleus	Nucleus	Nucleus	Nucleus
LOC_Os05g44260	*OsS40-2*	Nucleus	Mitochondria	Nucleus	Nucleus	Nucleus	Extra cell
LOC_Os10g27350	*OsS40-3*	Nucleus	Chloroplast	Chloroplast	------	Nucleus	Nucleus
LOC_Os03g02280	*OsS40-4*	Nucleus	Chloroplast	Chloroplast	------	Secretory	Extra cell
LOC_Os04g45834	*OsS40-5*	Nucleus	Chloroplast	Chloroplast	Nucleus	Nucleus	Nucleus, Extra cell
LOC_Os04g33760	*OsS40-6*	Nucleus	Nucleus	Chloroplast	Nucleus	Nucleus	Nucleus, Extra cell
LOC_Os01g52730	*OsS40-7*	Nucleus	Nucleus	Chloroplast	-------	Nucleus	Extra cell
LOC_Os10g33990	*OsS40-8*	Cytoplasm	Chloroplast	Chloroplast	Nucleus	Nucleus	Extra cell
LOC_Os01g64300	*OsS40-9*	Nucleus	Nucleus	Chloroplast	-------	Nucleus	Extra cell
LOC_Os01g52740	*OsS40-10*	Cytoplasm	Nucleus	Chloroplast	Nucleus	Nucleus	Nucleus, Extra cell
LOC_Os12g05980	*OsS40-11*	Mitochondria	Chloroplast	Chloroplast	Nucleus	Nucleus	Extra cell
LOC_Os11g05600	*OsS40-12*	Chloroplast	Chloroplast	Chloroplast	Nucleus	Nucleus	Extra cell
LOC_Os04g43990	*OsS40-13*	Chloroplast	Mitochondria	Chloroplast	---------	Chloroplast	Extra cell
LOC_Os05g45440	*OsS40-14*	Nucleus	Cytoplasm	Chloroplast	Nucleus	Nucleus	Nucleus
LOC_Os07g33270	*OsS40-15*	Nucleus	Mitochondria	Chloroplast	---------	Nucleus	Extra cell
LOC_Os07g32810	*OsS40-16*	Nucleus	Nucleus	Chloroplast	----------	Nucleus	Extra cell

* Publicly available web services YLoc, WoLF PSORT, DISTILL 2.0, LOCALI-ZER 1.0, BaCelLo and Euk-mPLoc-2.0 were used to predict the putative subcellular localization of *S40* proteins in rice.

**Table 3:** *Cis*-elements in the promoters of *S40* genes in barley, Arabidopsis, and rice.

*Cis*-Elements	*HvS40*	*HvSDI*	*HvSD8*	*At2g 34340*	*At1g 29640*	*At5g 45630*	*AtS4-3*	*OsS40-1*	*OsS40-2*	*OsS40-7*	*OsS40-14*
W-box	4	4	5	5	6	5	2	2	2	5	3
ERE	2									1	
MYB	19	3	2	1	2	6	7	5	3	5	6
LREs	13	11	7				2	1			
MYC	8	2	2	6	4	10	8	18	6	4	8
ABRE	6	5	4	4		15	15	6	4	8	4
Dof	5	19	7	11	18	12	26	4	12	8	5
PRE	5	15	11								
CGCG box	3	3	3			8			26	14	20
SURE	2	4	6	5		1		5	3	1	1
DRE/CRT	2	4	3		1	1		2	2	1	
LTR	2	2	3		1	1	1	3	2	3	
ARF	2		3	2		1		1			
DPBFCOREDCDC3	2		1	3		4	6	2	1	3	2
Pyrimidine box	1	3	2	1	2	1	2	1	3	1	
G-box plus G			1								

Promoter regions of 840, 1000, and 708 bp upstream of *S40* genes in barley, Arabidopsis, and rice respectively, were analyzed with the use of the PLACE program (Higo and others 1999). W-box: Binding site for WRKY TFs; ERE: Elicitor response element; MYB: Myeloblastosis; LREs: Light regulated elements; MYC: Myelocytomatosis; ABRE: Abscisic acid responsive elements; Dof: DNA-binding with one finger; PRE: Pathogen response elements; SURE: Sulfur response elements; DRE/CRT: Dehydration response elements/C-repeat; LTR: Low temperature response; ARF: Auxin response factor; DPBFCOREDCDC3: BZIP TFs binding core sequence; G-box plus G: TF OsIRO2-binding core sequence.
